# Humanized anti-hepatocyte growth factor (HGF) antibody suppresses innate irinotecan (CPT-11) resistance induced by fibroblast-derived HGF

**DOI:** 10.18632/oncotarget.4369

**Published:** 2015-06-08

**Authors:** Jong Kyu Woo, Ju-Hee Kang, BoRa Kim, Byung Hee Park, Kum-Joo Shin, Seong-Won Song, Jung Ju Kim, Hwan-Mook Kim, Sang-Jin Lee, Seung Hyun Oh

**Affiliations:** ^1^ College of Pharmacy, Gachon University, Incheon, Republic of Korea; ^2^ National Cancer Center, Goyang, Republic of Korea; ^3^ iBio, Inc., Seoul, Republic of Korea; ^4^ Yooyoung Pharmaceutical Co., Seoul, Republic of Korea

**Keywords:** hepatocyte growth factor (HGF), irinotecan (CPT-11), chemoresistance, microenvironment, colorectal cancer

## Abstract

The growth factors derived from the microenvironment create an environment conducive to tumor growth and survival. HGF deprivation using neutralizing antibody enhanced chemosensitivity in colorectal cancer cells (CRC). We determined secreted HGF in fibroblast conditioned medium (CM). Combination treatment of anti-HGF antibody and irinotecan (CPT-11) directly enhanced CPT-11 sensitivity in CRC. We generated xenograft in NOD/SCID mice inoculating HCT-116 human colorectal cancer cells subcutaneously with or without fibroblast. We found that the combination of CPT-11 and anti-HGF antibody induced marked suppression of tumor development. These results suggest that HGF produced by fibroblast induce CPT-11 resistance, and that anti-HGF antibody abrogate such resistance *in vivo*. fibroblast-derived HGF is important determinant of chemoresistance. Anti-HGF monoclonal antibody treatment confirmed the importance of this growth factor for chemoresistance in CRC. These results present new options toward the early diagnosis of chemoresistance and suggest novel combinations of chemotherapy and anti-HGF agents to prevent or significantly delay the onset of therapy resistance.

## INTRODUCTION

Hepatocyte growth factor (HGF), also known as scatter factor, is a pleiotropic polypeptide growth factor with a number of biological activities, including cell scattering, stimulation of cell motility, mitogenesis, morphogenesis, angiogenesis, and cellular invasiveness [1–3]. HGF was identified in 1984 [4, 5] and was subsequently determined to be a heterodimeric molecule composed of an alpha and beta chain [6]. The importance of HGF in organ development is demonstrated by HGF null mutation mice, which exhibit embryonic lethality [7]. *In vivo*, HGF and its receptor, the c-MET proto-oncogene product, are thought to be involved in embryogenesis, tissue reorganization, and tumor progression [8]. Overproduction of HGF by tumor cells or tumor-associated stromal cells and increased expression of the c-Met protein are two mechanisms that contribute to aberrant stimulation of this pathway. Consequences of dysregulated HGF and c-Met expression include tumor cell migration, proliferation, and protection from apoptosis [9]. Overexpression of HGF and c-Met has been detected in numerous cancer types and is associated with a worse prognosis [10].

A bilateral collaborative effort of normal epithelial cells and components of the stromal compartment maintain the integrity of a normal physiological system. The tumor microenvironment contains a diverse array of cell types, including endothelial cells, pericytes, smooth muscle cells, macrophages, mesenchymal stem cells and stromal fibroblasts, among others [11–13]. The stromal cells were crosstalk not only with tumor cells but with each other [14]. Tumorigenesis has classically been viewed as a largely cell-autonomous process involving genetically transformed cancer cells. Recently, many investigations support the notion that tumor stromal cells play important roles in tumor initiation, progression, and metastasis [15–17]. Fibroblasts are the most frequent component of tumor microenvironment, especially in colorectal, breast, ovarian and pancreatic cancers [18, 19]. Fibroblasts are often identified by their spindle-shaped morphology, their ability to adhere to plastic, and their lack of markers indicating other cell types [20]. Cancer associated fibroblasts (CAFs) stimulate malignant cell proliferation by providing different types of growth factors and cytokines in a context-dependent manner [21], such as HGF [22], members of the epidermal growth factor family [23], fibroblast growth factor (FGF) [24], Wnt families [25], and IL-6 [26]. CAFs are phenotypically and functionally distinguishable from their normal counterparts in their increased rate of proliferation. CAFs secrete a wide variety of growth factors, chemokines, collagens, and matrix-modifying enzymes, collectively supplying a communication network and altered three-dimensional ECM scaffold that governs the proliferation of cancer cells, tumor invasion, and metastasis across tissue types [27].

Despite the large repertoire of therapies available and the continuing efforts to incorporate new drugs into clinical practice, innate and acquired resistances remain intractable problems in clinical care. While the investigation of therapeutic resistance has not surprisingly centered on tumor cell-intrinsic mechanisms to date, recent findings have uncovered novel roles for the tumor microenvironment in modulating therapeutic efficacy [28]. Cancer cells are in contact with their surrounding cells by paracrine and autocrine mechanisms. The identification of resistance mechanisms has revealed a recurrent theme-the engagement of survival signals redundant to those transduced by the targeted kinase [29]. It is possible that evaluation of the HGF/c-MET signaling axis in the tumor may indicate the susceptibility of that tumor to show resistance through this pathway [30]. Indeed, higher expression of the c-MET receptor in different cancer cell lines correlated with their resistance to targeted therapy stimulated by HGF [31–33]. Based on these premises, the purpose of this study is to understand the role of fibroblasts-derived HGF in mediating the chemoresistance phenotype of cancer cells and the mechanism by which this occurs.

In the present study, we demonstrated that HGF secreted from fibroblast promotes chemoresistance of CRC. Thus, we have focused on evaluating the clinical significance of HGF/c-Met axis in cancer chemotherapy. The identification of stromal HGF as a mediator of CPT-11 resistance has important translational potential. In addition to this, we investigated the effects of anti-HGF monoclonal antibody on cancer chemoresistance.

## RESULTS

### CM from colonic fibroblast rescues colorectal and lung cancer cells from growth suppressive activity of CPT-11

**Figure 1:**
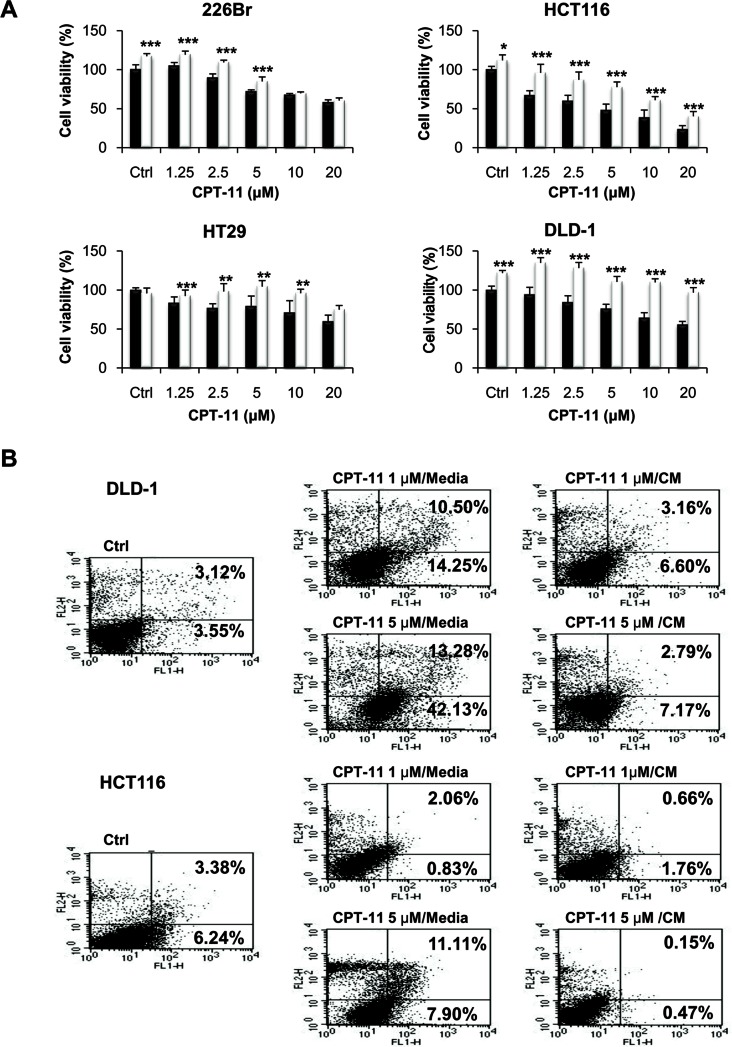
Conditioned media (CM) from fibroblasts enhances CPT-11 resistance in cancer cells **A.** Human lung and colorectal cancer cells were plated into 96-well plates at a density of 3 × 10^3^ cells/well on day 0. Under serum free culture media (black bar), cells were incubated with CCD-18co colonic fibroblast CM (white bar) and/or CPT-11 for 48 h. Inhibition of cell proliferation was determined by MTT assay. Significant differences were evaluated using an unpaired two-tailed Student's *t*-test. (Error bars denote the standard deviation [SD]) (*p < 0.05, **p < 0.01 and ***p < 0.001). **B.** Induction of apoptosis in colorectal cancer cells. Cells were stained with PI and Annexin V-FITC, followed by flow cytometry analysis. Cells were treated with CPT-11 under serum free media or CCD-18co colonic fibroblast CM. The apoptotic cells were determined by Annexin V-FITC positive staining.

We postulated that innate drug resistance might be caused at least in part by factors secreted by the tumor stromal cells. While growth and metastasis-promoting effects of the stromal cells have been well reported [34], a role in drug resistance has only been partially described [35, 36]. To determine if the rescue effect was activated by the secretion factors, we tested the ability of CM from fibroblasts to recapitulate the resistance effect. For this purpose, cancer cells were treated with CPT-11 at concentrations ranging from 1.25 to 20 μM with or without CM from fibroblasts for 48 h. As shown in Figure 1A, cell proliferation was significantly suppressed by CPT-1 in all the human cell lines as compared with the vehicle control. Under these experimental conditions, cells became highly resistant to CPT-11 in the presence of CM from fibroblasts. CM from fibroblasts rescued human cancer cells from CPT-11, indicating that the rescue was due to a factor secreted by the fibroblasts. Similar results were obtained when we determined apoptosis of cells by annexin-V/PI staining after incubation of cells with CPT-11 in the presence of CM from fibroblasts (Figure 1B). CPT-11 caused apoptosis in DLD-1 and HCT116 cells; however, CM from fibroblast significantly attenuated apoptotic cell death in response to CPT-11. Figure 1 indicates that CM from fibroblasts enhanced the resistance against CPT-11 in cancer cells and suppressed apoptotic cell death, indicating that secreted factors from fibroblasts are crucial for sustaining cancer cell survival.

### HGF derived from fibroblasts activates c-Met receptor in cancer cells

**Figure 2:**
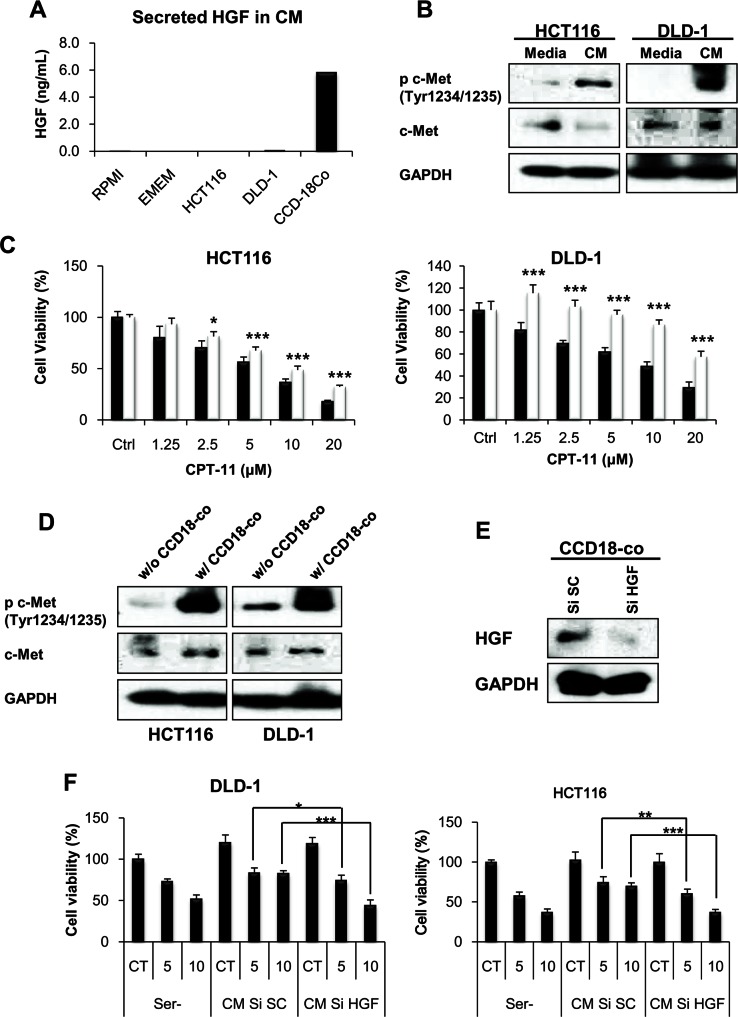
fibroblast-derived HGF activates c-MET receptor and induces CPT-11 resistance in colorectal cancer cell **A.** HGF secreted by cancer cells (HCT-116 and DLD-1) and colonic fibroblasts (CCD-18co) were measured. Cells were cultured with serum free medium for 24 h and HGF concentrations were determined by ELISA. **B.** CM from fibroblast activates c-MET receptors. HCT-116 and DLD-1 cells were cultured with serum free media or CCD-18co CM for 1 h. Cells were collected, and the indicated proteins were detected by western blotting. **C.** Colonic fibroblast cells promote CPT-11 resistance of colorectal cancer cells (HCT-116 and DLD-1). Cancer cells were cultured with (white bar) or without (black bar) CCD-18co cells, in the presence or absence of CPT-11 (1.25-20 μM) for 48 h, and inhibition of cell proliferation was determined by MTT assay. Significant differences were evaluated using an unpaired two-tailed Student's *t*-test. (Error bars denote the standard deviation [SD]) (*p < 0.05 and ***p < 0.001). **D.** Co-culture with colonic fibroblast CCD-18co cells increases c-MET receptor activation in colorectal cancer cells. HCT-116 and DLD-1 cells were co-cultured with CCD-18co cells for 24 h. Lysates were analyzed for c-MET activation by western blotting. **E.** Inhibition of HGF production from fibroblast by transfection with HGF siRNA. Colonic fibroblast cells were transfected with 10 nM HGF siRNA or scramble siRNA. After transfection, cells were collected and lysates were submitted to Western blotting to quantify HGF. **F.** HCT-116 and DLD-1 cells were cultured with CM from HGF siRNA transfected fibroblast for 48 h in the presence or absent of CPT-11. Cell viability was determined by MTT assay. Significant differences were evaluated using an unpaired two-tailed Student's *t*-test. (Error bars denote the standard deviation [SD]) (*p < 0.05, **p < 0.01 and ***p < 0.001).

To determine whether fibroblast-derived HGF is responsible for CPT-11 resistance, we measured the amount of HGF in the CM from fibroblasts. HGF is not expressed in epithelial cells and therefore c-Met receptor requires HGF production by surrounding stromal cells for ligand-dependent activation [37]. As shown in Figure 2A, the ELISA assay demonstrated that HGF production is only shown in CM from fibroblast. The c-Met receptor tyrosine kinase activation induces pleiotropic biological effects in a wide variety of cells, including mitogenic, motogenic, morphogenic, and anti-apoptotic activities [10, 38]. To determine whether fibroblast-derived HGF activates the c-Met receptor resulting in CPT-11 resistance, we treated cells with CM from fibroblasts. We detected c-Met activation by treatment of fibroblasts-derived CM (Figure 2B). To mimic the tumor microenvironment, we performed co-culture experiments with fibroblasts and cancer cells. We investigated whether fibroblasts in co-culture adopted compensatory mechanisms such as the activation of the c-Met receptor and increased resistance to CPT-11 by cancer cells. As shown in Figure 2C and 2D, co-culture with fibroblasts induced resistance to CPT-11 and activated the c-Met receptor in cancer cells. To determine whether HGF is directly implicated in the activation of c-Met and the resistance to CPT-11, we knocked-down HGF by siRNA and measured cell viability in the presence of CPT-11. The HGF siRNA significantly suppressed HGF expression in CCD-18co cells (Figure 2E). As expected, CM from HGF siRNA treated cells failed to rescue cancer cells from the apoptosis by CPT-11 (Figure 2F). These results confirm the importance of fibroblast-derived HGF in CPT-11 resistance of cancer cells and indicate that HGF might be a therapeutic target for overcoming resistance to CPT-11.

### Targeting of fibroblast-derived HGF abrogates HGF stimulated CPT-11 resistance in colorectal cancer cells

**Figure 3:**
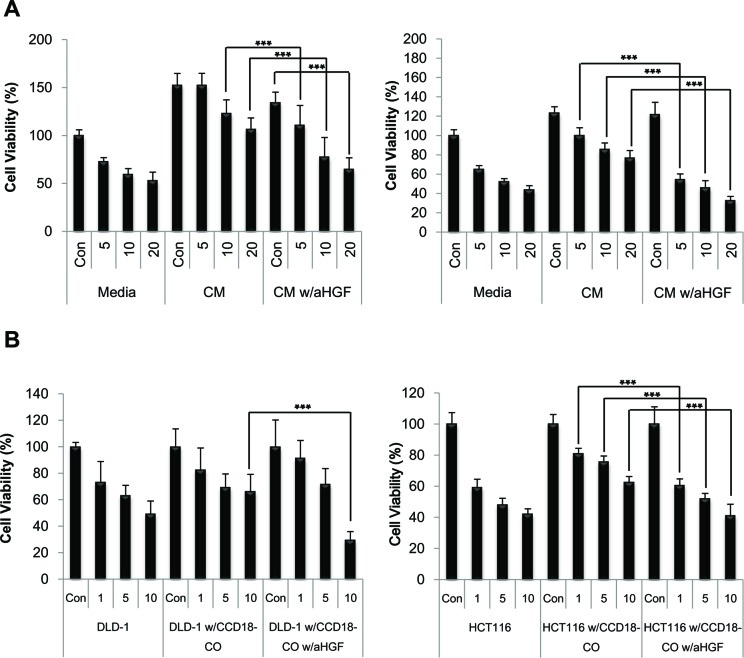
Humanized anti-HGF antibodies attenuated HGF activated c-Met signaling pathway and enhanced apoptotic cell death induced by CPT-11 in colorectal cancer cells **A.** HCT-116 (right panel) and DLD-1 (left panel) cells were treated with or without CPT-11 and/or CCD-18co CM in the presence or absence of humanized anti-HGF antibody (200 ng/ml) for 48 h. **B.** HCT-116 (right panel) and DLD-1(left panel) cells were cultured with CCD-18co cells with or without CPT-11 in the presence or absence of humanized anti-HGF antibody (200 ng/ml) for 48 h. The inhibition of cell proliferation was determined by MTT assay. Significant differences were evaluated using an unpaired two-tailed Student's *t*-test. (Error bars denote the standard deviation [SD]) (***p < 0.001).

To test whether HGF directly contributes to the effect on overcoming CPT-11 resistance in cancer cells, anti-HGF antibody was added to the CM to neutralize the HGF activity. The viability of cancer cells in the CM from fibroblasts was significantly decreased by the addition of 200 ng/ml of the anti-HGF antibody (Figure 3A). To determine the inhibitory effect of anti-HGF antibody in conditions mimicking a tumor microenvironment, co-cultures of fibroblasts and cancer cells were subjected to MTT assay (Figure 3B). As shown in Figure 3B, neutralization of fibroblast-derived HGF maintained the reduction in cell viability induced by CPT-11 indicating that HGF targeting significantly enhances CPT-11 stimulated anti-cancer activity. Taken together, our findings suggest that the fibroblast-derived HGF may play an important role in cancer cell survival and that the interference with its effect in tumor cells may represent a novel strategy for the treatment of colorectal cancers.

### Humanized anti-HGF antibody attenuated HGF activated c-Met signaling pathway and enhanced apoptotic cell death induced by CPT-11 in colorectal cancer cells

**Figure 4:**
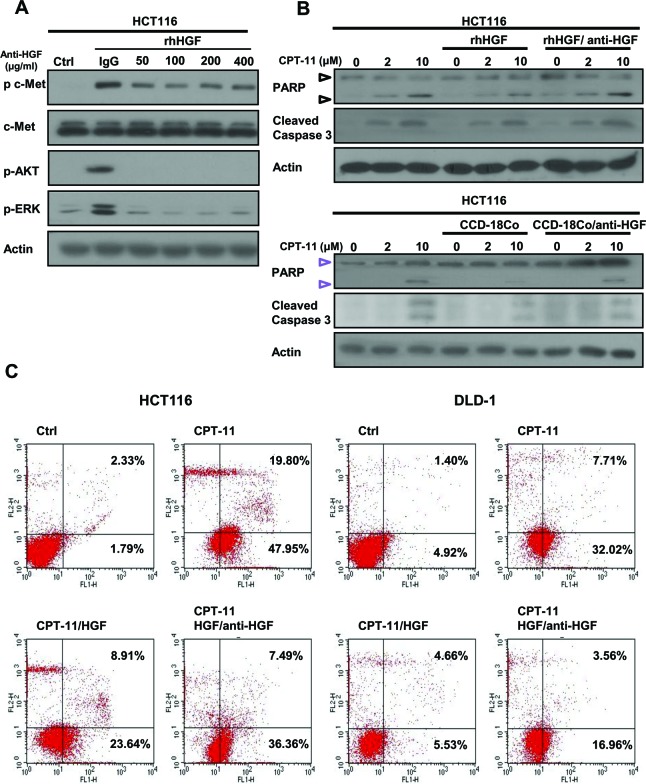
Humanized anti-HGF antibodies inhibit HGF-activated c-MET signaling pathways and enhanced apoptotic cell death induced by CPT-11 **A.** Humanized anti-HGF antibody inhibited c-MET signaling pathways. HCT-116 cells were treated with or without recombinant HGF (50 ng/ml) in the presence or absence of humanized anti-HGF antibody for 24 h. Cells were lysed, and the indicated proteins were detected by western blotting. **B.** Protein lysates were collected and subjected to western blotting to detect pro-apoptotic molecules such as cleaved PARP and cleaved caspase-3. **C.** Cells were stained with PI and Annexin V-FITC followed by flow cytometry analysis. The apoptotic cells were determined by Annexin V-FITC positive staining.

To examine the effects of anti-HGF antibody on cell signaling and apoptosis in cancer cells, we performed Western blot analysis. Anti-HGF antibody (200 ng/ml) effectively suppressed c-Met, AKT, and ERK activation induced by recombinant HGF (50 ng/ml) in HCT116 cancer cells (Figure 4A). Anti-HGF antibody (200 ng/ml) enhanced cleavage of PARP and caspase-3 which are apoptotic molecules induced by CPT-11 (Figure 4B upper panel). Anti-HGF antibody also enhanced cleavage of PARP and caspase-3 induced by CPT-11 in HCT116 cells co-cultured with CCD-18C human colon fibroblast cells (Figure 4B, lower panel). These results indicate that HGF-induced CPT-11 resistance is activated by HGF/c-Met signaling and is abrogated by treatment with anti-HGF antibody. Furthermore, we examined whether anti-HGF antibody augmented apoptotic cell death inhibited by recombinant HGF. We found that CPT-11 induced significant apoptotic cell death, recombinant HGF inhibited that apoptosis, and anti-HGF antibody reversed such inhibition. For example when cells were treated with CPT-11 alone, 5 μM of CPT-11 increased apoptotic cell death in HCT116 and DLD-1 cells by 67.75 % and 39.73 %, respectively. When cells were treated with recombinant HGF after treatment with 5 μM CPT-11, apoptotic activity was suppressed by 32.55 % and 10.19 % in HCT116 and DLD-1, respectively. The rescue effect stimulated by recombinant HGF was attenuated by anti-HGF antibody (200 ng/ml) (43.85 % and 20.52 % in HCT116 and DLD-1, respectively). These results indicated that CPT-11 resistance is induced by fibroblast-derived HGF/c-Met signaling and is attenuated by treatment with anti-HGF antibody in colorectal cancer cells.

### Fibroblast-derived HGF induces CPT-11 resistance in cancer cells and anti-HGF antibody restores the sensitivity of tumor growth to CPT-11 *in vivo*

**Figure 5:**
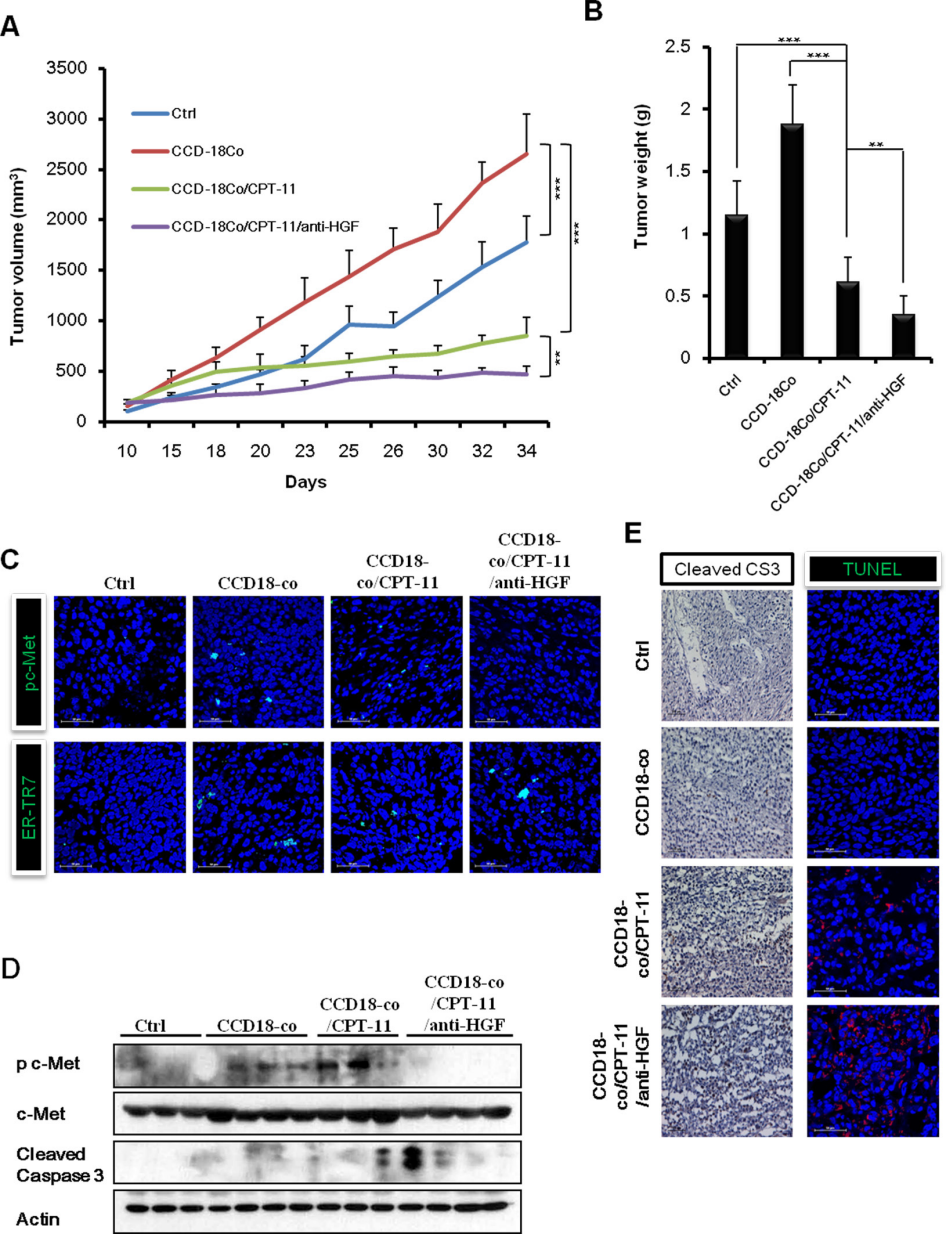
Combination of humanized anti-HGF antibody and CPT-11 inhibits tumor growth *in vivo* HCT-116 cells with or without CCD-18co cells were inoculated subcutaneously into NOD/SCID mice Mice in the control group of received PBS. Mice in the treatment groups received CPT-11 (10 mg/kg; 3 times/wk; intraperitoneally) with or without humanized anti-HGF antibody (10 mg/kg; 2 times/wk; intraperitoneally). **A.** Tumor volume was measured every 3 or 4 days and calculated. **B.** Average tumor weights were measured on day 34. The differences were statically significant between the combination treatments versus single treatment. Significant differences were evaluated using an unpaired two-tailed Student's *t*-test. (Error bars denote the standard deviation [SD]) (**p < 0.001 and ***p < 0.001). All four treatment groups were well tolerated, as total mouse body weights remained unchanged (<10 % fluctuation). **C.** Immunofluoresence images of phospho-c-Met receptors and a fibroblast marker (ER-TR7) in tumors. Co-staining with DAPI was done to show nuclei. Frozen tumor specimens were subjected to immunofluorescence analysis using anti-phospho-c-Met antibody (green; upper panel) or anti-ER-TR7 antibody (green; lower panel). **D.** Western blot analysis for cleaved caspases-3 and phospho-c-Met receptors. **E.** Immunohistochemistry and TUNEL assays were performed to observe apoptotic cell death in tumors. Immunohistochemical analysis in tumors was performed using an antibody against cleaved caspase-3 (left panel). Tumor sections were stained with hematoxylin for counterstaining. The red fluorescent staining indicates positive apoptotic cells and DAPI staining was used as a nuclear counterstaining (right panel).

To examine the possible induction of CPT-11 resistance by fibroblast-derived HGF and the efficacy of combined treatment with anti-HGF antibody and CPT-11 on colorectal cancer *in vivo*, we generated xenografts by inoculating HCT116 human colorectal cancer cells, with or without fibroblast CCD-18co cells, into NOD/SCID mice subcutaneously. We found that the mice injected with HCT116 plus CCD-18co cells had tumors that grew significantly faster than those in mice injected with HCT116 cells alone. CPT-11 treatment suppressed the growth of tumors in mice grafted with HCT116 plus CCD-18co cells. Interestingly, the combination of CPT-11 and anti-HGF antibody treatment induced the strongest suppression of tumors. The *in vivo* tumor growth analysis showed that injecting mice with HCT116 and CCD-18co cells led to significantly faster tumor growth as compared with only injecting HCT116 cells (2653.04 ± 396.62 mm^3^ versus 1775.46 ± 257.53 mm^3^ *p* < 0.001). Marginal suppression was observed with CPT-11 treatment (1775.46 ± 257.53 mm^3^ versus 850.38 ± 183.15 mm^3^, *p* < 0.05). The combination treatment with CPT-11 plus anti-HGF significantly inhibited tumor growth as compared to the control group, HCT-116 plus CCD-18co group and the HCT-116 plus CCD-18co treated with CPT-11 groups (1775.46 ± 257.53 mm^3^ versus 467.57 ± 81.90 mm^3^, *p* < 0.001; 2653.04 ± 396.62 mm^3^ versus 467.57 ± 81.90 mm^3^, *p* < 0.001; 850.38 ± 183.15 mm^3^ versus 467.57 ± 81.90 mm^3^, *p* < 0.05, respectively). At the end of the experiment, tumor weight in the control, HCT-116 plus CCD-18co, HCT-116 plus CCD-18co with CPT-11, and HCT-116 plus CCD-18co with CPT-11 plus anti-HGF groups were 1.15 ± 0.27 g, 1.88 ± 0.31 g, 0.61 ± 0.20 g and 0.35 ± 0.15 g, respectively. These results suggest that anti-HGF antibody abrogates fibroblast-derived HGF-induced CPT-11 resistance *in vivo* (Figure 5A and 5B). To determine whether anti-HGF antibody decrease HGF/c-Met signaling, we examined the expression of phospho-c-MET by immunofluorescence and western blotting (Figure 5C and 5D). Our data revealed that c-Met signaling was suppressed in the anti-HGF antibody treatment group when compared to HCT-116 plus CCD-18co and HCT-116 plus CCD-18co with CPT-11 groups. In addition, a fibroblast marker (ER-TR7) was detected in HCT116 plus CCD-18co tumors. Whereas, the tumors in mice injected with HCT116 alone did not produce a detectable level of ER-TR7 (Figure 5C). As expected, tumors in the anti-HGF antibody and CPT-11 treatment group contained a significantly higher number of TUNEL positive and cleaved caspase-3 positive cells as compared with HCT-116 plus CCD-18co and HCT-116 plus CCD-18co with CPT-11 groups (Figure 5D and 5E). Taken together, these results suggests that cancer associated fibroblasts could change the nature of cancer cells and generating resists to chemotherapy.

## DISCUSSION

Irinotecan (CTP-11) is a semi-synthetic analog of camptothecin, which was originally isolated from *Camptotheca acuminate* [39]. CTP-11 inhibits topoisomerase-1 (Topo-I) activity by trapping Topo-I-DNA cleavage complexes, leading to lethal replication-mediated double strand breaks [40]. CPT-11 is used as a first- and second-line chemotherapy for advanced or recurrent colorectal cancer and has shown activity in patients with advanced colorectal cancer resistant to leucovorin (LV) and 5-fluorouracil (5-FU) [41, 42]. Although treatment of advanced CRC patients with CPT-11 as a single agent has shown response rates of approximately 25 %, these rates can reach 60 % when used in combination with other agents [43, 44]. However, the efficacy of CPT-11 is strongly limited by the development of drug resistance. Classically, drug resistance may be due to modulated drug influx/efflux, gene amplification and subsequent overexpression of the target molecule or alteration in drug target specificity drug detoxification by increased levels of drug target, enhanced DNA repair in the case of DNA-damaging drugs or NF-kB activation [39]. Recent studies have proposed that microenvironment could be associated with chemoresistance [45]. Stromal cells could potentially induce chemoresistance acquisition in tumor cells, including cell-cell and cell-matrix interactions, local release of soluble factors and so on [46–48]. We hypothesized that fibroblasts generate soluble factors that could stimulate resistance to chemotherapy. The fibroblast cell line CCD-18co conditioned medium can support the survival of colorectal cancer cells against CPT-11. Using co-culture experiments *in vitro* and *in vivo*, we found that a minority of CCD-18co cells could rescue the colorectal cancer cells from CPT-11 treatment.

These results indicate that a novel strategy, targeting the crosstalk between tumor cells and stromal fibroblasts, may be important for circumventing the drug resistance of colorectal cancers. By using colonic fibroblast cell line CCD-18co and chemotherapy drug CPT-11, we demonstrate that fibroblasts play a functional role in chemoresistance of colorectal cancer cells presumably through fibroblast-derived HGF in tumors via c-MET signaling. Our results revealed that CPT-11 resistance of colorectal cancer cells were enhanced upon exposure to CCD-18co CM and co-culture with CCD-18co cells *in vitro*. Furthermore, CCD-18co cells produced and secreted HGF in CM and c-Met (receptor tyrosine kinase of HGF) was activated upon exposure to CCD-18co CM. HGF neutralizing antibody effectively reduces the activation of HGF/c-MET signaling, which blocks its ability to promote CPT-11 resistance in colorectal cancer cells. Our finding indicates that HGF is involved in CRC chemoresistance. The administration of anti-HGF antibody significantly reverses the chemoresistance effect of fibroblast-derived HGF *in vivo* and *in vitro*.

Multiple biological actions of HGF, mediated via c-Met tyrosine phosphorylation, are outlined. These activities depend on downstream adaptor molecules recruited by c-Met tyrosine-phosphorylated multi-docking sites [10]. These phosphotyrosines stimulate high-affinity interactions with various src homology region 2-containing cytoplasmic effectors, including Gab1, PI3-kinase, Shc, and Grb2. We demonstrate here that HGF activates Akt and ERK signaling pathways which inhibited apoptosis processing in CRC. Different studies suggest that HGF triggers anti-apoptotic signals [49–51]. The present study demonstrates that fibroblast-derived HGF can protect cells from apoptosis by means of both Akt and ERK signaling. Our data indicate that fibroblast-derived HGF rescues cells from apoptosis and that the anti-HGF antibody sensitizes cells to apoptosis and abolishes HGF protection.

When human colorectal cancer cells were inoculated with colonic fibroblast cells in immunodeficiency mice, they enhanced drug resistance to CPT-11. This finding is consistent with other reports that have shown that acquired resistance to chemotherapy reagents is a problem [52–54]. Indeed, our research has shown that anti-HGF antibody can reverse the fibroblast-derived resistance to CPT-11. This phenomenon, besides highlighting the importance of the tumor microenvironment, further confirms that fibroblast-derived HGF/c-MET signaling is involved in resistance to chemotherapy reagents. In our *in vivo* models, the combination of CPT-11 with anti-HGF antibody showed a potent anti-cancer effect on human colorectal cancer, as evidenced by the strong inhibition of tumor growth and interference with c-MET signal transduction. We also demonstrated that combination treatment was significantly associated with the induction of apoptotic cell death. Consistently, our research suggests that the combination treatment of CPT-11 with anti-HGF could interfere with the process of acquired. In summary, HGF appears to play a substantial role in the complex mechanisms involved in the induction of chemoresistance of colorectal cancer by fibroblasts.

In summary, we identified fibroblast-derived HGF activated CPT-11 resistance through c-MET signaling pathways. Our findings point to the micro-environment as an important factor but this does not preclude the likely involvement of other growth factors and/or cytokines. Moreover, our results suggest that such resistance mechanisms can be uncovered through the systematic dissection of interactions between tumors and their micro-environment. Future studies should therefore seek to identify such resistance mechanisms for all of the drugs that are in development, potentially leading to mechanism-based combination regimens such as the CPT-11 and c-MET inhibitor combination proposed here.

## MATERIALS AND METHODS

### Cell culture and reagents

The human colorectal cancer cell lines, HCT116, HT29 and DLD-1 and lung cancer cell line 226Br were cultured in RPMI1640 (Welgene, Seoul, Korea) containing 10% fetal bovine serum (FBS; Welgene) and antibiotics (100 mg/L penicillin and 100 mg/L streptomycin; GIBCO-BRL Life Technologies; Gaithersburg, MD, USA). Human colonic fibroblasts (CCD-18co) were cultured in DMEM (Welgene, Seoul, Korea) containing 10 % FBS and antibiotics Cells were incubated in a humidified thermostat under 5 % CO_2_ at 37 °C. A predesigned siRNA targeting HGF and scramble siRNA were purchased from Shanghai Genepharma (Shanghai, China). Recombinant Human HGF was purchased from R&D system (Minneapolis, MN, USA). Humanized anti-HGF antibody was kindly provided by YooYoung pharmaceutical Co. Ltd (Seoul, Korea) and iBIO Inc (Seoul, Korea). Irinotecan (CPT-11) was obtained from Sigma Aldrich (St. Louis, MO, USA). Antibodies to c-MET, phosphorylated-c-Met, phosphorylated-ERK, phosphorylated-AKT, PARP, and cleaved Caspase-3 were purchased from Cell Signaling Technology (Beverly, MA, USA). Antibody against HGF, actin and fibroblast marker (ER-TR7) were purchased from Santa Cruz Biotechnology (Santa Cruz, CA, USA).

### Conditioned medium preparation

Conditioned medium (CM) were generated from 70-80 % confluent CCD-18co human colonic fibroblasts cultured with serum free RPMI1640 for 48h. All cellular debris was removed by filtration (0.4 μM). The level of human HGF in CM was determined by using Human HGF ELISA KIT (R&D system, Minneapolis, MN, USA) according to the manufacturer's protocol. For HGF knockdown studies, early-passage CCD-18co cells were transfected with 10 nM siRNA using Lipofectamine 2000 (Invitrogen, Carlsbad, CA, USA) according to the manufacturer's recommendations. Conditioned medium from HGF siRNA transfected CCD-18co cells were used to assess colorectal cancer cell proliferation assays.

### Assay for cell proliferation

Cells were seeded into 96-well plates (SPL, Seoul. Korea) and cultured 24 h before exposure for 48 h to various concentrations of CPT-11, recombinant HGF, or anti-HGF antibody, as indicated. Following this, 20 μl of 3-(4,5-dimethyl-thiazol-2-yl)-2,5-diphenyltetrazolium bromide (MTT, Sigma) solution (5 mg/ml) was added to each well and the plate was incubated at 37 °C for 2 h. The medium was then discarded, and the purple-blue MTT formazan precipitate was dissolved in 100 μl dimethyl sulfoxide (DMSO). The plates were mixed and absorbance at 570 nm was measured by an ELISA reader (Bio-Rad Laboratories, Hercules, CA, USA).

### Western blotting

Western blotting was performed, as described previously [55]. Briefly, whole-cell lysates were prepared in a modified RIPA buffer containing proteinase inhibitors and phosphatase inhibitors, as described elsewhere [56]. Equivalent amounts of protein (20-80 μg) were loaded onto 10 % or 12 % sodium dodecyl sulfate-polyacrylamide gel electrophoresis (SDS-PAGE) gels and transferred by blotting to polyvinylidene fluoride membranes. The blot was incubated with primary antibodies for over nigh at 4 °C and then Horseradish peroxidase-conjugated secondary antibodies for 2 h at room temperrature. The protein-antibody complexes were detected using enhanced chemiluminescence (Amersham, Arlington Heights, IL, USA), according to the manufacturer's recommended protocol.

### Apoptosis analysis

Apoptosis was determined by FACS analysis using propidium iodide (PI) and Annexin-V-FITC (Santa Cruz, CA, USA). Briefly, cancer cells were plated in 6 well plates with or without CM derived from CCD-18co cells. Cells were treated with different concentrations of CPT-11 with or without anti-HGF antibody the next day. After 48 h, cells were collected, re-suspended in binding buffer, and incubated with PI and Annexin-V-FITC for 10 min at 4 °C. Cells were then analyzed by flow cytometry, using a FITC signal detector with an excitation wavelength of 488 nm and an emission of 530 nm (to detect Annexin-V -FITC), and a PI signal detector with an excitation wavelength of 496 nm and an emission of 615 nm (to detect PI). We defined “apoptotic cell death” as the population of cells that were positive for annexin-V-FITC staining.

### *In vivo* xenograft models

For xenograft model, 5-6 week-old NOD/SCID mice (NOD/LtSz-Prkdcscid/J) were purchased from KRIBB (Cheongwon-gun, Korea). HCT116 human colorectal cancer cells 5 × 10^6^ in 100ul of PBS were injected subcutaneously (s.c.) in the left flank. HCT116 human colorectal cancer cells 4.5 × 10^6^ and 5 × 10^5^ CCD-18co cells were injected in the right flank. Tumor volumes were calculated as length × width^2^ × 0.5. Mice were weighed and tagged and when the tumor reached the standard volume of approximately 200 mm^3^, the mice was randomly assigned to four experimental groups (n = 5): Control; CPT-11treatment; humanized anti-HGF Ab treatment; CPT-11/humanized anti-HGF Ab combination treatment. CPT-11 (10 mg/kg, three times a week) and humanized anti-HGF (40 mg/kg/day, twice a week) were administered intra-peritoneally for 3 weeks. Tumors were measured twice weekly using digital calipers. All animal experiments were approved by the Institutional Animal Care and Use Committee of the National Cancer Center.

### Immunohistochemistry and TUNEL staining

The tumors were fixed in 10 % neutral buffered formalin and embedded in paraffin. Immunohistochemical assays and terminal deoxynucleotidyl transferase-mediated UTP end-labeling (TUNEL) staining was done as previously described [57]. For immunohistochemistry, sections were incubated with cleaved caspases-3 antibodies overnight at 4 °C. Antibody detection was performed using the EnVision detection system (Vector Laboratories, Burlingame, CA, USA) according to the manufacturer's protocol. Another part of the tumors was sectioned for TUNEL staining for the measurement of apoptosis. TUNEL assay was performed as previously described [57]. For apoptosis analysis, we counted all nuclei in the field as well as all TUNEL-positive cells in three to five randomly selected fields of view at a magnification of ×200.

### Statistical analysis

All experiments were done in duplicate or triplicate. A two-tailed Student's *t*-test was used for statistical analysis of comparative data using Microsoft Excel software. Values of *p* < 0.05 were considered statically significant and indicated by asterisks in the figures. For graphical representation of data, y-axis error bars indicate the standard deviation (s.d.) of the data for each point marked on the graph.
